# “I was afraid to go to the hospital”: A qualitative analysis and ethical implications of the impacts of COVID-19 on the health and medical care of older adults in Ethiopia

**DOI:** 10.1177/20503121241263305

**Published:** 2024-07-30

**Authors:** Kirubel Manyazewal Mussie, Jenny Setchell, Mirgissa Kaba, Bernice Simone Elger

**Affiliations:** 1 Institute for Biomedical Ethics, University of Basel, Basel, Switzerland; 2 School of Health and Rehabilitation Sciences, The University of Queensland, Brisbane, QLD, Australia; 3 School of Public Health, Addis Ababa University, Addis Ababa, Ethiopia; 4 Center for Legal Medicine, University of Geneva, Geneva, Switzerland

**Keywords:** Ageing, COVID-19, prioritization, healthcare access, distributive justice, ethical issues

## Abstract

**Objective:**

The COVID-19 pandemic has brought severe health consequences among older adults and posed ethical challenges. The aim of this study was to explore the impacts of COVID-19 on the health and medical care of older adults in Ethiopia and associated ethical implications, regardless of older adults’ COVID-19 infection status.

**Methods:**

In this qualitative study, we followed an inductive exploratory approach based on reflexive thematic analysis. We conducted semistructured interviews between March 2021 and November 2021 with 20 older adults and 26 health professionals who were selected from healthcare facilities and communities in Ethiopia using purposive and snowball sampling techniques. We audio-recorded, transcribed, translated, and inductively analyzed the interviews using thematic analysis.

**Results:**

Participants reported that the pandemic compromised the accessibility and quality of both COVID and non-COVID healthcare services for older adults, which negatively impacted older adults’ health conditions and medical care. Moreover, participants elaborated on the health conditions and care of older patients with COVID-19 and highlighted that older COVID-19 patients often have severe health conditions, do not get adequate COVID-19 care, and may receive lower priority for admission to intensive care units compared to younger patients when resources are limited.

**Conclusions:**

Results of this study showed that practices of COVID-19 care and measures may have led to adverse consequences such as limited availability and access to aged care in Ethiopia, which could have further health consequences on older patients. Our results contribute to a better understanding of ethical issues such as distributive justice and prioritization arising in the healthcare of older patients in times of global pandemic. It is imperative for local and international health policymakers and ethicists to further analyze and address the challenges that compromise the accessibility and continuity of quality care for older persons during a public healthcare crisis.

## Introduction

Coronavirus disease 2019 (COVID-19) is an infectious disease caused by a virus named severe acute respiratory syndrome coronavirus 2 (SARS-CoV-2) that has rapidly spread and overwhelmed health systems worldwide following its first discovery in December 2019. A report from the Word Health Organization (WHO) showed that as of 11 February 2024, there had been 774,631,444 confirmed cases and 7,031,216 deaths globally due to COVID-19.^1^ Older adults are at the highest risk of hospitalization and death from COVID-19 due to chronic health conditions such as hypertension and diabetes, which weaken the immune system and increase susceptibility to severe complications from the virus.^2,3^ Moreover, various lockdown measures also had psychosocial impacts on older adults such as loneliness and isolation.^4^ The progress and impact of COVID-19 in Africa were generally perceived as relatively less severe compared with the situation in Global North, due to factors such as a younger population demographic, previous exposure to other infectious diseases providing some level of immunity, and swift public health responses.^5,6^ However, experts widely agree that the pandemic resulted in severe public health consequences in the region and there are increasing concerns about its ongoing impacts on the continent.

The health impact of COVID-19 on older adults is a critical concern for the African region where the current older population (60 and above), which is already 62 million, is rapidly increasing and expected to triple in the coming three decades.^7^ Although the proportion of older adults in the Global South is lower in comparison to countries in the Global North,^3^ the lack of proportional and quality aged care for the older population in developing countries is a critical public health challenge^8^ that raises ethical concerns such as distributive justice and lack of access to healthcare (the earlier referring to, more broadly, the fair distribution of resources, opportunities, and benefits within a society).^9,10^ This is especially true in the time of a global pandemic which greatly affected fragile healthcare systems^11,12^ and dramatically increased mortality among older adults in several African countries. This increase was due to, among other things, limited access to healthcare.^13^ These challenges continue to be a critical concern in Ethiopia where, as of 11 February 2024, there had been 501,060 confirmed cases of COVID-19 with 7574 deaths.^1^ This is particularly worrying for the country’s increasingly ageing population, which was 5.2 million (approx. 5% of the total population) in 2015 and is expected to be total 8.4 million by 2030.^14^ The health and wider socio-economic challenges of the pandemic have persisted^4,15,16^ despite various measures such as physical distancing, self-isolation, and contact tracing taken at the time by the government to fight the disease.^17–19^

The support system for older adults in Ethiopia could be described as complex. On the one hand, “family care is a centuries-long tradition,”^20^ and the care of older people is highly dependent on a traditional support system based on family and community welfare. On the other hand, however, such traditional support structures have been rapidly weakening due to, for example, urbanization and the death or migration of persons who were sources of support for older adults.^4,20^ Thus, the lives of several older adults are put at stake as they are positioned between dwindling traditional support on the one hand and, on the other, a lack of a healthcare system that is sensitive to the needs of older adults. The Ethiopian government has been taking some steps since the 1990s to ensure the health and well-being of older adults in the country. The Developmental Social Welfare Policy issued in November 1996—which was then replaced by the Social Protection Policy in November 2014^21^ and the 10-year National Plan of Action on Older Persons developed in June 2006^22^ are two government policy instruments to date that were tailored to meet the needs of older adults. In particular, the overarching goals of the 10-year National Plan of Action on Older Persons include enhancing community-based services for older adults, ensuring their rights and interests are embedded in poverty reduction strategies, and fostering collaboration between government and non-governmental organizations to address issues affecting older persons.^22^ However, these efforts have been insufficient to meet public health needs^21^ and several studies report that as a result of an inadequate system of support and protection, many older people in Ethiopia continue to suffer health and wider socio-economic problems.^9,23–25^

These challenges became more critical and have warranted increased attention in the era surrounding the COVID-19 pandemic which has affected health, social, economic, and political facets of life. Putting extraordinary pressure on the country’s health systems that was already burdened with other infectious diseases,^26–29^ the pandemic posed unprecedented ethical challenges in relation to, for example, healthcare access, decision-making, resource limitation, and prioritization. For reasons such as their age and general health conditions, older adults were (and continue to be) a key group of people affected by such ethically problematic situations created by the pandemic. For example, the use of age (prioritizing younger patients) as one of the criteria for patient triage concerning access to lifesaving resources was a debatable practice during the height of the pandemic that continues to be ongoing practice in different healthcare contexts.^30,31^ Even though there is limited evidence in Ethiopia about this specific experience, existing literature from Ethiopia indicates that patient triage in the face of health resource limitations pose ethical concerns such as fairness and distributive justice.^32,33^

There is little research exploring, and providing the ethical implications of, the impact of COVID-19 on the health of older adults and the medical care they receive in Ethiopia. Those studies that do exist suggest that there are concerning ethical issues for older Ethiopians. For example, one cross-sectional study^16^ conducted in southern Ethiopia reports that COVID-19 affected the health-related quality of life of older patients most as compared with other population groups. Similarly, three quantitative studies^34–36^ assessing the severity of COVID-19 symptoms among COVID-19 patients identify that older patients suffer more serious consequences, such as severe illness and death, than other age groups. Additionally, a qualitative study^4^ explored the socioeconomic consequences such as poverty and loneliness of COVID-19 lockdown measures among Ethiopian older adults. None of these studies specifically explored and provided an ethical analysis of the impacts of COVID-19 on the health and medical care of older adults. Additionally, a recent scoping review on ethical issues in aged care in Eastern Africa reports a lack of evidence addressing ethical challenges in aged care in the region including Ethiopia.^37^ Thus, the aim of this study was to explore the impacts of COVID-19 on the health and medical care of older adults in Ethiopia and associated ethical implications, regardless of older adults’ COVID infection status.

## Methods

### Design

In this qualitative study, we used reflexive thematic analysis, employing an inductive, exploratory approach as the study context has not yet been investigated in-depth.^38–40^ This methodological approach allowed us to gather detailed data and explore experiences and perspectives related to the pandemic without being bound by a specific theoretical framework. Thus, while our study did not rely on a particular theory, the inductive approach facilitated a nuanced understanding of the topic and allowed for the reflexive exploration of complex phenomena within the context of the COVID-19 pandemic and its impact on older adults in Ethiopia. This study was approved by the Ethics Committee of the University of Basel in Switzerland (approval no. UEK024520) and the City Government of Addis Ababa Health Bureau in Ethiopia (approval no. 071211-AAHB). Written informed consent was obtained from all participants before the study. This qualitative study followed the Consolidated Criteria for Reporting Qualitative Research (COREQ) guideline for qualitative studies.^41^

### Participants

Participants were both older adults (*n* = 20) and health professionals (*n* = 26) selected using purposive and snowball sampling techniques and based on eligibility criteria.^42,43^ The definition of old age used in this study is 60 years and above, which is taken from the UN definition of older persons for sub-Saharan African countries^14^ and is also in line with the formal retirement age of 60 in Ethiopia.^22^ Thus, the inclusion criteria for the older adults were being 60 years old or above, having a current and/or past medical treatment experience in Ethiopia, and being capable of communicating and giving informed consent.^44^ The inclusion criteria for health professionals involved being a consenting individual currently employed in a healthcare facility, with either current or past experience in providing formal healthcare to older adults aged 60 and above in Ethiopia. This participant group included professionals such as nurses, medical doctors, and internists. Both older adults and health professionals were recruited from four government and two private healthcare facilities in Addis Ababa, Ethiopia’s capital. The government healthcare facilities included Tulu Dimtu Health Center, Bole 17 Health Center, Menelik Medical Center, and Black Lion Hospital, while the private facilities were the International Cardiovascular and Medical Center and the Nordic Medical Center. The healthcare facilities were chosen considering their level (from primary to tertiary levels) and ownership (government and private) as such diversity helps to provide a broader range of perspectives when little knowledge exists on a topic.^45^

All participants were contacted and recruited in person by KMM and with the assistance of medical directors at selected facilities who also verbally approved the request for participant selection. Participants were informed about the researcher’s professional background, including their qualifications and experience in conducting qualitative research. Personal goals or reasons for conducting the research were not disclosed to prevent potential bias or influence on participants’ responses. The consent process with all participants was conducted with an emphasis on maintaining arm’s-length relationships and ensuring freedom from potential coercion. Prior to participating, all interviewees were provided with hard copies of written informed consent forms along with all information about the study. Additionally, the interviewer reiterated the study purpose and the contents of the written consent form, asking participants to rephrase their responses to ensure full comprehension of what they were consenting to. In some cases, particularly with interested participants, this was done a few days before the interviews, which further helped in establishing a professional relationship and rapport with the participants. Then, all participants returned the signed written informed consent and the researcher conducted the interviews in person. Participation in this study was voluntary, and participants did not receive compensation as they did not incur any costs due to their participation.^46,47^ The total number of participants in this study was 46 (26 health professionals and 20 older adults), which was determined based on the iterative nature of our data analysis process.^48^ We continuously reviewed and analyzed the data as it was collected, allowing us to assess when there is sufficient depth and repetition of concepts in the data to provide a rich understanding of the study aim. At this stage, we concluded that the sample size was sufficient to achieve a diverse range of perspectives and to obtain a comprehensive understanding of participants’ experiences and perceptions.

### Data collection

We conducted semi-structured interviews with a total of 46 participants between 03 March 2021 and 28 November 2021. The interviewer (KMM) was a male PhD student at the time of the interviews and had prior training and hands-on experience in qualitative interviewing techniques. During the data collection phase, potential biases arising from the interviewer’s academic and cultural background were addressed by discussing them with project members. This approach aimed to mitigate bias and ensure the integrity of the data. All interviews were conducted at the selected healthcare facilities, in private rooms where no one else than the interviewer and participant was present. The interviews were conducted in Amharic, the official language of Ethiopia and the interviewer’s first language. Two pilot interviews—one with an older adult and another with a health professional—were conducted to ensure that the interview guides were understandable to participants and that the questions were open-ended enough to facilitate discussions.^49^ In response to the feedback, minor changes were made by, for example, avoiding or elaborating medical terms, finding more appropriate Amharic translations of ethical concepts, and broadening opening questions to facilitate probing and further discussions. Adhering to the standard practice in in-depth semi-structured interviewing,^50^ the interviews were conducted in an open-ended and conversational manner. The first author prepared the interview guides based on discussions with project supervisors. The interview guide was broader than only COVID-related questions, but for this study, we have only analyzed questions aimed at capturing the perspectives of older adults and health professionals on: (1) how the pandemic affected the accessibility of quality healthcare services to older patients during the pandemic, (2) the health consequences of COVID-19 on older adults and the adequacy of COVID-19 treatment services, and (3) the level of priority given to older patients with COVID-19 when resources are limited (see Additional file 1). Each interview took approximately 1 h on average (range: 15–107 min). All interviews except two were audio-recorded upon verbal consent from participants. Two participants did not consent to be voice recorded and so the interviewer took written notes about these participants’ responses. The recordings (in Amharic language) were transcribed verbatim into English and anonymized by KMM. The transcripts were checked for quality and translation accuracy by MK and KMM.

### Data analysis

The data were entered into MAXQDA-2022 software to store transcripts and facilitate the analysis process.^51^ The transcripts were not returned to participants for comment or correction, mainly due to resource limitations and a commitment to minimize any potential distress or discomfort associated with revisiting sensitive topics. However, the research team implemented rigorous methods during data analysis, including thorough checks for transcription and translation accuracy, to enhance the trustworthiness of the findings. We followed Braun and Clarke’s reflexive thematic analysis approach consisting of six phases: familiarizing with the data, generating initial codes, searching for themes, reviewing themes, defining and naming themes, and producing the report.^52^ While employing this approach, we also drew from Braun and Clarke’s recent critical reflection on the use of thematic analysis for better rigor.^53^ Thus, acknowledging that “TA (thematic analysis) refers not to a singular approach, but rather to a cluster of sometimes conflicting approaches,” we specifically followed the reflexive TA approach by which coding is open and themes are the final outcome of the analysis.^53^ In light of this and using the inductive content analysis approach,^54^ KMM initially performed an open coding of the data with inputs from all co-authors. Based on comments from the remainder of the research team on the initial coding stage, KMM read through the transcripts again, revised the codes, and categorized them into tentative themes. Then two authors (KMM, JS) revised the codes and KMM produced the final coding tree. The analysis resulted in two themes and five subthemes, which were finally agreed upon by all authors. Any disagreements were included in the analysis.

## Results

The analysis of semi-structured interviews with 20 older adults and 26 health professionals resulted in two themes and five subthemes that explore the impact of COVID-19 on the health of older adults and the medical care they receive. The themes are summarized in Table 1 and are presented below with illustrative quotes.

**Table 1.** Themes and subthemes.

Themes	Subthemes
Ethical issues relating to the health impacts of COVID-19 on older adults regardless of their COVID-19 status	Limited access to healthcare
	Compromised quality of care
The health conditions of older adults with COVID-19 and gaps in their medical care	Health conditions of older adults with COVID-19
	Inadequate COVID-19 treatment

Participants’ demographic characteristics are presented in Table 2. The older adults were aged between 60 and 87 years, with most being in the younger age range (only 3 were over 80 years). The health professionals were predominantly nurses (46.1%) followed by general practitioners (19.2%), internists (11.5%), public health officers (7.7%), and other professionals.

**Table 2.** Demographic information of participants.

Participant ID	Age	Gender: M (male) or F (female)	Underlying health conditions*	Occupation
Older adults				
OP1	65	F	Chronic leg injuries, stomach ulcer, diabetes	No
OP2	86	M	Joint pain, hypertension, urinary tract infection	No
OP3	63	F	Diabetes, glaucoma	Self-employed
OP4	87	M	Joint pain, lung problem (continuous coughing)	No
OP5	75	F	Thyroid disease, hypertension, vision problem, diabetes	No
OP6	68	F	Diabetes, hypertension, joint pain	No
OP7	63	F	Hypertension, hypertension, cardiovascular diseases	No
OP8	84	M	Joint pain, gastritis, chronic kidney disease, eye problem	No
OP9	74	F	Joint pain, stomach ulcer	No
OP10	64	F	Stomach ulcer	Physician
OP11	76	F	Leg injury, hypertension, joint pain	No
OP12	65	M	Backpain, diabetes	Self-employed
OP13	62	F	Diabetes, hypertension, joint pain	Self-employed
OP14	64	F	Migraine	Self-employed
OP15	61	F	Joint pain	Pastor
OP16	60	M	Diabetes, stomach ulcer	Self-employed
OP17	75	M	Joint pain, chronic kidney disease	No
OP18	69	M	Liver disease, gallbladder stone, hypertension	No
OP19	60	M	Backpain, migraine	Factory worker
OP20	77	M	Joint pain, thyroid disease	No
Total: 20 older adults				
Participant ID	Age	Gender: M (male) or F (female)	Profession	Number of years of work experience as a health professional
Health professionals				
HP1	26	F	Nurse	3
HP2	36	F	Nurse	9
HP3	46	M	Cardiologist	19
HP4	43	M	Health Officer	15
HP5	56	F	General Practitioner	23
HP6	61	F	Nurse	31
HP7	62	M	Neurologist	27
HP8	31	F	Internist	4
HP9	27	F	Nurse	3
HP10	32	F	Internist	6
HP11	36	F	Nurse	8
HP12	29	F	Nurse	2
HP13	31	F	Internist	5
HP14	28	F	Nurse	4
HP15	38	F	Nurse	7
HP16	26	F	Nurse	1
HP17	34	F	Nurse	5
HP18	31	M	Nurse	4
HP19	30	M	General Practitioner	6
HP20	32	F	Psychiatrist	5
HP21	36	M	General Practitioner	7
HP22	38	M	Public Health Officer	11
HP23	35	M	Nurse	13
HP24	31	F	General Practitioner	4
HP25	44	M	Public Health Officer	16
H26	25	M	General Practitioner	1
Total: 26 health professionals				

* The underlying health conditions of older adult participants were as reported by the older adult participants themselves in the interviews and not taken from hospital records.

### Theme 1: Ethical issues relating to the health impacts of COVID-19 on older adults regardless of their COVID-19 status

Our participants frequently expressed that COVID-19 has affected the health of older individuals in Ethiopia beyond direct infection with the disease. They indicated that the pandemic could influence the health of older adults irrespective of whether they contracted COVID-19. These effects were categorized into two main areas: limited access to healthcare and poor quality of healthcare.

#### Limited access to healthcare

Participants commonly discussed the broad effects of the COVID-19 pandemic on older adults’ access to healthcare services. Health professionals first noted that *older adults are considered as one of the risk groups for COVID-19 (HP11)*. For this reason, according to a few participants, older adults often avoided visiting healthcare facilities when they needed healthcare services. Similarly, some older participants said that accessing healthcare was difficult not because the services are inaccessible per se, but because they are risky and they chose not to visit. Referring to the time when the fear of COVID-19 was at its peak in Ethiopia, one older patient said, *at that time when I was sick I was afraid to go to the hospital, so I took drugs personally (OP3).* Thus, the fear of contracting COVID-19 was reported to cause older adults to engage in practices such as self-prescription of medications that were usually provided by a professional, with potentially severe health consequences. On the contrary, one older adult participant mentioned that they would go to a hospital for a routine follow-up: *Unless it is time to come for my appointment of the thyroid, I do not want to come. This hospital is like a marketplace. The crowd is not good because I may catch corona since they treat all in one place. (OP5)* The fear of infection among older adults reflects a significant barrier to accessing essential medical services. As the above quotes illustrate, this fear is not merely a matter of personal preference but is rooted in systemic issues. For instance, healthcare facilities may be overcrowded, increasing the risk of exposure to COVID-19. Additionally, the lack of separate treatment areas for older patients exacerbates their concerns about potential infection.

Another reason for not seeking healthcare, as one participant added, relates to community discussions about COVID-19 measures in healthcare settings:

OP3: “I heard people saying if you have high fever and sickness, they will take you inside into the COVID room. I heard that they will force you to get in and make you fill out their COVID statistics. Therefore, I have not gone to health centres for 4 or 5 months.”

I: “When you stayed away from there [the hospital], did you experience any health consequences?”

OP3: “Yes, I became seriously sick. I had a severe headache at that time. Even if I wanted to go there, because I heard that, even from my neighbors, saying they force you to go in as a corona suspect, I was afraid. If I went to the health centre and started a follow-up back then, my blood pressure would be identified and they would prescribe me the medicines.”

Similarly, health professionals voiced their concerns for older adults who seek healthcare during the pandemic. It was argued that *the pandemic has definitely affected them [older adults in general] with regards to admission and treatments and so on (HP12)*. Healthcare facilities were occupied with COVID-19 patients and *there was more shortage of patient beds than before (HP15).* Since their health conditions often make older adults vulnerable to the disease, one health professional underlined, *they have the fear of the disease, and they worry about what will happen to them and would be careful not to be infected (HP15)*. In support of this, another health professional added:

Generally, when you say older people, usually what you fear is about those with comorbidity. And if hospitals are saturated with COVID centers, where are we going to take these older patients? The beds which were occupied by non-COVID patients are now occupied by COVID patients. And a non-COVID patient cannot stay with a COVID patient in one room. So, because of such reasons, older patients are at home and yes they are being affected. (HP12)

The health professional highlighted that hospitals overwhelmed by COVID-19 cases often lack space for older patients with comorbidities. Due to COVID-19 patients occupying beds originally designated for non-COVID cases, older patients are forced to stay at home, resulting in limited access to healthcare services. Participants gave numerous examples of the many serious impacts of this reduced access, such as kidney dialysis patients not receiving their regular care, and cancer patients’ reviews being delayed.

#### Compromised quality of care

A few of the participating health professionals said that they believe the pandemic has affected the quality of healthcare older adults receive. The participants’ opinions presented under this subtheme are about care services provided for older adults regardless of their COVID infection status. As mentioned above, a few health professionals mentioned that some older patients with regular treatment needs were receiving less frequent care than pre-pandemic: *These days we don’t provide our older patients with as regular appointments. But by looking at the expiry date of the medicines, we schedule their appointments to be 6-monthly or so. (HP4)* Even though health professionals said that they used other ways to compensate for this gap such as telephone follow-ups, the absence of in-person check-ups was reported to have affected the older patients’ medical progress. Due to COVID-19 preventive measures such as physical distancing and shorter medical consultations, health professionals were not able to connect with patients with nonverbal communication in the way they used to.

When the older patients come and then go happily, I won’t just sit here and say goodbye. Instead, I go with them to the pharmacy and watch them until they leave the compound. You know what? They even look at me as their son and touch my hair, even though I am bald [laughs]. But nowadays there is this corona disease that forbids us from physical contact. So, for the sake of their health, you keep your distance from them. You make them not want to touch you. To miss someone means communicating and wanting to be with that person. They miss you when they have good communication with you, and want to make contact with you. (HP4)

In addition, one health professional posited that some older patients and their families do not want to come to their medical appointments even when requested by health professionals. This participant said that families of these older patients sometimes refused to take them to hospitals out of fear that the older patients might be infected with COVID-19. The participant considered this to affect both the older person’s health condition and medical progress:

When the older patients come here, they have already deteriorated. Even though the dialysis is done, it will be too late. Those who needed dialysis were in a situation where they have to get extra medical attention because they had missed the previous appointment. There have been so many cases like this. (HP21)

The quote illustrates the delayed access to critical medical interventions like dialysis for older patients due to the impact of COVID-19. By the time older patients reach healthcare facilities, their health has significantly deteriorated, leading to missed appointments and the need for additional medical attention. This highlights the collateral health impacts of the pandemic on older patients, exacerbating their existing health conditions and reducing their access to timely and essential healthcare services.

Additionally, health professional participants reported that older patients admitted to hospitals expressed dissatisfaction because resources are mostly occupied by COVID-19 patients and, for example, they had to wait long in the queue to get medical service. One health professional specifically highlighted that some older patients could be very unhappy and complain to authorities.

We had an incident where we had a COVID suspect patient [not an older patient]. We placed the patient on oxygen support since he was having difficulty breathing. [. . .] You cannot place an older patient near this patient who is having COVID symptoms. We were in dilemma. We tried to explain the situation but the older patient was saying “I was here first for treatment” and complained saying we took his time and disrespected him. (HP1)

The participating health professionals gave further examples of reactions such as verbal scolding and reporting to authorities among older patients when non-COVID care services were limited. These experiences such as complaints from older patients (regardless of their COVID status) and cancellation of hospital appointments are indications that services are reduced in quality and may therefore impact health outcomes.

### Theme 2: The health conditions of older adults with COVID-19 and gaps in their medical care

#### Health conditions of older adults with COVID-19

This theme encompasses common responses by the majority of the health professional study participants discussing what they perceive to be the health conditions of older adults directly related to COVID-19 infections. More specifically, old age and comorbidities were reported to not only increase the chance of infection but also worsen symptoms among older patients when compared with younger COVID-19 patients: *Unlike the young patients, older patients have co-morbidities such as diabetes and blood pressure and this makes COVID strong on them. (HP16)* One health professional added the following to further elaborate this:

Basically when you get old your reserve is poor for different reasons. From birth starting from day one we are ageing. Your kidney reserve is bad, your lung will likely have underlying issues, and in your heart, you probably have hypertension or some cardiac issues. And all in all, COVID is a bad disease that affects each and every system. (HP8)

Other health professionals discussed further complexities such as reduced immunity and increased dependency on others for care (e.g., support to eat, toilet). However, as a few health professionals noted, they believed that not every older patient who tests positive for COVID-19 dies or experiences severe symptoms: *I had a 96-year-old COVID patient who said to me ‘doctor, it [COVID-19] missed me’. Nothing happened to him and he did not even require admission. (HP8)*. This implies exceptions to the severe consequences of COVID-19 infection on older patients. While the overarching theme of the study is the impact of COVID-19 on the health and medical care of older adults, this specific theme delved into how health professionals perceive the health conditions of older adults with COVID-19. The quotes provided shed light on the unique challenges faced by older patients, including the exacerbation of symptoms due to comorbidities and age-related factors, such as reduced immunity and increased dependency on others for care. The discussion aims to capture the complexities of COVID-19’s impact on older adults’ health, contributing to understandings of the broader gaps in medical care for this vulnerable population during the pandemic.

#### Inadequate COVID-19 treatment

Some health professionals specifically commented on the quality of healthcare older patients with COVID-19 receive. Although the lack of adequate COVID-19 treatment might have affected all COVID patients in general, as our participants’ responses imply, older patients could have been disproportionately affected as they became more seriously ill and required more treatment. COVID-19 patients in general could be admitted to different levels of healthcare—primary, secondary, and tertiary—depending on the severity of their symptoms. This could be either through the referral system—meaning by being referred from a primary health facility to a tertiary, for example—or through direct admission for severe cases.^55^ The quality of COVID-19 care was reported to be different from one healthcare level to the other. For example, as one health professional noted, older patients with COVID-19 in the tertiary hospital where the participant works often expressed unwillingness to return to primary healthcare facilities due to the poor treatment they had experienced there: *They [older patients] said things like ‘I will not go because if I go there, they would kill me.’ (HP14)* Another health professional further elaborated on their perceptions of the quality of COVID-19 treatment by making specific reference to inequitable treatment in relation to the economic status of older patients with COVID-19.

There were many older people with COVID living on the street and there were many ethical questions like whether older people with low economic status will have equal protection as compared to the higher 1%. I am still not aware if the care they have received, for example like the ones in charity-based nursing homes, is the same as the ones getting treated in good hospitals. (HP12)

There were also discussions about differences in terms of the quality of COVID-19 care between government and private healthcare facilities. It was often argued that private facilities provide better care. One participant summarizes this,

As far as I know there is nothing I can mention as a problem on the care older patients with COVID receive, especially if we talk about our hospital as compared to other facilities. But if we talk about other facilities yes, there are older patients with COVID who received inadequate care and came here. [. . .] So, there are people who come to us in a bad state because they did not get good service in other places. Government hospitals have heavy patient flow and resource scarcity. So, it is a bit challenging there. (HP11)

This was echoed by other health professionals from similar private settings, such as a participant who said *this is a fancy place, but in government health facilities this is not available (HP8).* Even though health professionals from government healthcare facilities did not compare government with private facilities in terms of adequate availability of treatments in their interviews, they generally commented on the challenges they perceived in government settings to have implications on the quality of COVID-19 treatment. For example, one health professional said, *with the kind of limited facilities and availability of professionals that we have in government hospitals, the queue is long and older patients need to wait. And with the presence of this COVID, it gets a little tougher. (HP1)* One older patient had similar thoughts: *compared with government hospitals, the private ones have better treatment even though they are expensive. (OP4)* Overall, our participants’ comments suggested that there was better healthcare quality for older patients with COVID in private facilities, and there were no further comments from participants regarding the difference between private and government facilities to elaborate the gaps among private health facilities.

#### Low priority for older patients with COVID-19

Within the theme of care for older patients with COVID-19, another subtheme we developed relates to the concept of prioritization, which was discussed by the health professional participants only. Participating health professionals expressed that they often have difficulty addressing the care needs of all COVID-19 patients with the limited human and material resources available. Thus, they said that they often needed to prioritize among COVID-19 patients and this usually ended up disadvantaging older patients. A few health professionals who discussed the challenges of prioritizing among COVID-19 patients reported that they would use age as one of the criteria: *One thing you look at when prioritising is age. The chance of giving attention to an 80 or 90-year-old is low when there is a 20-year-old struggling to breathe. (HP8)* However, none of these participating health professionals mentioned that their decision to use age as one of the criteria for patient triage was based on written guidelines. How much weight they give to age as a criterion differed between these participants. For example, one health professional noted that age alone cannot be used as a criterion and that there are other things to consider when deciding which COVID-19 patient should get treatment first.

The things that will be an issue are usually with regards to oxygen demand and machines. Since we are very tight with resources, the decisions about who to prioritize are dependent on the kind of cases that come. When there are patients that need a machine or oxygen supply, we prioritize those. So, it is the cases that mostly decide who is prioritized. It is difficult to base it just on age. If the patient is critical, even if he/she is an older patient or not, it is how critical they are that is prioritized. (HP14)

On the other hand, some other health professionals’ responses suggested that they relied heavily or solely on age to triage access to COVID-19 treatment. Thus, from these discussions, younger COVID-19 patients were seemingly prioritized regardless of predicted outcomes: *if a 30-year-old COVID patient has more comorbidities and less chance of survival than a 90 or 87-year-old COVID patient, I will definitely lean toward saving the young patient (HP12).* Nevertheless, age was reported to be a relevant determining factor when all other conditions are the same between two COVID-19 patients.

The participating health professionals who discussed patient age as an important criterion for accessing COVID-19 care also listed some reasons for prioritising older patients less. As one participant said, one reason was the perceived low chance of survival among older patients, *if you see the 90-year-old person [referring to a COVID-19 patient], his chance of surviving is questionable (HP18).* A second reason two participants mentioned was the argument that an older person has already lived long and enjoyed life while a younger one has not yet: *Quite honestly, after 70 years old that COVID patient is probably not working. He did the things you have wished to do when you get old. What is left for him is touring the world and having fun. (HP12)* Another participant’s response supports this perspective by referring to cultural expressions and values:

If their [a young and an older patient] health conditions are similar, you then look at the age difference. At least the older one has lived. They say “he has eaten well through all these ages.” Even the older patient himself, if he were able to communicate, he would not want to be treated first if he sees a younger person at that same time unless he is a selfish person. (HP18)

A final reason a few more participants highlighted is the assumption that older patients will not live long to contribute to society, while younger patients will. One health professional said, *I would give priority to the young one because the young are in a state of benefiting us in the future. The older one has served a lot already. I think it is better to think about the future than the past. (HP16)* Another participant added, *But for a 30-year-old person, their life is just beginning. They may be newly married and have a one-year-old child. [. . .] So, for me it is easier to give up on the older patient and resuscitate the younger patient first. (HP12)* These participants provided other similar examples and argued that prioritizing younger patients with COVID-19 over older patients has more benefits to immediate family members of the patient in particular as well as to the wider community in general.

## Discussion

This study used an inductive approach to explore the health consequences of COVID-19 on older patients in Ethiopia, substantiating a broader body of COVID-19 research in different contexts.^15,34,56–59^ The findings of this study contribute toward a better understanding of ethical concerns arising in the healthcare of older patients in times of such a global pandemic. Our research highlights that there are many ethical issues implicit in the COVID-19 response in Ethiopia relevant to older patients, although most may not be new but have not been previously explored in this country. Analyzing interview data collected from 26 health professionals and 20 older adults, our results suggest that due to the pandemic, (1) the availability and quality of healthcare services for older patients are likely to have been compromised regardless of their COVID-19 infection status, (2) older patients with COVID-19 might have received inadequate COVID-19 treatment and be at risk of being de-prioritized for COVID-19 treatment in favor of younger patients. These factors raise key ethical concerns that require attention and debate.

Our first theme highlighted the perception among our interviewees that the unexpected crisis brought by the novel coronavirus pandemic exacerbated gaps in healthcare access and quality for older people in Ethiopia. Older adults seeking non-COVID health services were reportedly frequently unable or unwilling to access them for reasons such as the pandemic’s load on healthcare resources and fear of COVID infection. The fear of contracting COVID-19 significantly impacted older adults’ willingness to seek healthcare services, as highlighted by both health professionals and older participants. This fear stemmed from the perceived risk associated with visiting healthcare facilities during the pandemic, leading to practices such as self-prescription of medications to avoid potential exposure. This finding reflects an ethical issue because it highlights disparities in access to healthcare and raises questions about equitable treatment. Older adults should have equal access to medical care without fear of potential harm based on their age. Moreover, the systemic factors contributing to this fear, such as overcrowded healthcare facilities and inadequate safety measures, underscore broader ethical concerns related to healthcare resource allocation and patient safety. Additionally, community discussions about COVID-19 measures in healthcare settings further fuelled apprehension, with concerns raised about being labelled as a COVID-19 suspect upon seeking care. Health professionals echoed these concerns, emphasising the strain on healthcare facilities due to the influx of COVID-19 patients, which resulted in a shortage of patient beds and resources for non-COVID cases. Consequently, older patients with comorbidities faced challenges accessing necessary healthcare, contributing to adverse health outcomes such as delayed treatments for conditions like kidney dialysis and cancer. These observations underscore the complex interplay between fear, healthcare accessibility, and the broader impacts of COVID-19 on older adults’ health and medical care.

Our results suggest that this likely resulted in treatment and follow-up interruptions. Such interruptions are known to have serious health consequences in old age, such as increased hospitalization and mortality.^60,61^ Similar to our results, a recent scoping review on healthcare access and service utilization among older adults reports that due to the pandemic, older patients in several countries such as India and the USA had limited access to non-COVID related services such as medications for non-communicable diseases and other chronic medical conditions.^62^ Further, several studies^63–66^ in Ethiopia have shown that the pandemic has affected the provision of essential, non-COVID health services to the population across age groups, which has put many lives at risk. According to one quantitative study, the use of inpatient and outpatient care in Ethiopia dropped by 20% and 27%, respectively, after the pandemic.^63^ Another cross-sectional study reports that Ethiopian non-COVID medical services were reduced by 50% after the pandemic.^65^ Similar challenges were recorded in other African countries.^5,67^

Our second theme implied that older patients with COVID-19 could have similar challenges accessing COVID-19 treatment. Even though other studies suggest that COVID-19 service preparedness and treatment are generally low in Ethiopia,^15^ our findings further suggest that health services specific to COVID-19 were likely even more unresponsive to the healthcare needs of older patients. Our research suggests that the pandemic may have disproportionately and severely affected the health of older adults. This finding is consistent with quantitative studies in other countries such as China,^58^ Malaysia,^68^ Italy,^69^ Spain,^70^ Nigeria,^71^ and several African countries.^72^ Despite this evidence, other literature suggests that COVID-19 treatment services were often not adequately accessible to older patients.^73,74^ Our findings from health professionals imply that older patients with COVID-19 were less prioritized and might not have enough access to COVID-19 treatment.

Our study suggests that the COVID-19 pandemic raises several ethical challenges arising mainly in relation to resource limitation and priority setting. Lack of healthcare access is an ethical issue per se,^75^ especially when vulnerable population groups such as older patients are involved.^76^ In line with this, the WHO states that “from resource allocation and priority-setting, physical distancing, public health surveillance, [. . .], the COVID-19 pandemic presents serious ethical challenges.”^77^ Ethiopia, being one of the most resource-scarce countries with a per capita gross national income of USD 890 in 2020,^78^ has encountered similar challenges that raise ethical issues such as inappropriate distribution of health resources and health inequalities. The suggestions of age-based decision-making reported in our study have several implications for the ethical debates surrounding ageism in healthcare, also since age is often identified as a strong risk factor for COVID-19 hospitalization and death.^31,79^ While its use as a direct criterion for rationing is rejected in some existing guidelines and laws in situations where resources are scarce, for example, insufficient number of ventilators or to be transplanted organs,^80–82^ there are a few voices stirring some controversy.^83,84^ In relation to this, our findings are consistent with existing evidence on age-based intensive care unit (ICU) decision-making at the beginning of the pandemic in Italy.^85^ When there is a shortage of treatment equipment, especially during an emergency public health crisis such as COVID-19, it is a common practice to consider different patient characteristics such as case severity, chance of survival, and so on, when determining which patient should get priority. However, the international consensus is that prognosis should be used and not age as such (age playing a role only indirectly as one of the factors influencing prognosis).^80–82^

Our results suggest that some health professionals might be making potentially ageist decisions where older COVID-19 patients with a better chance of survival might be de-prioritized over younger patients with less chance of survival. However, such decisions accord with the fair innings argument which “maintains that for healthcare resources to be distributed fairly every person should receive sufficient healthcare to provide them with the opportunity to live in good health for a normal span of years.”^86^ Thus, this principle makes age an important criterion when triaging patients and favors younger patients with the ultimate goal of giving them the chance to live (at least somewhat) longer. On the contrary, WHO’s Working Group on Ethics and COVID-19 adds that it is essential not to discriminate against older patients in times of resource scarcity:

[. . .] this pandemic appears to significantly impact older adults (those 60 years of age or older), and such characteristics are relevant to shaping priorities for the allocation of resources during COVID-19. As a result, it may be inappropriate to use critical care triage guidelines that have age cut-offs that deprioritize or exclude those aged over 60 years.^87^

The health system in Ethiopia often faces challenges in priority setting, and the lack of adequate guidelines to facilitate clinical decision-making about, for example, bedside rationing and end-of-life issues have ethical implications for distributive justice and equity in healthcare.^32,33,88^ A study on patient priority setting in Ethiopia conducted among physicians a few months before the pandemic began summarizes pre-pandemic challenges: “Physicians have few or no written guidelines or policies to instruct them on how to prioritize delivery of care when need exceeds supply; a first come, first served strategy is often used.”^33^ In addition to revealing the use of this strategy, data from Ethiopia indicate that physicians likely employ other criteria such as prognosis, age, and working condition.^33,89^ For example, the majority of physicians in one survey study conducted before the pandemic reported they would prioritize children over both older patients and patients who are economic providers in the family.^33^

To our knowledge, prioritizing by age is not a recommendation in any policy or guideline in Ethiopia but appears to occur in practice. The Federal Ministry of Health (FMOH) devised a COVID-19 management manual to address, among others, the challenges of decision-making when demands exceed resources. An ethics section of this document acknowledges the challenging ethical issues arising from COVID-19 and states some principles to address concerns.

During the care of patients with COVID-19 at facilities, many ethical issues are expected to arise in the clinical care process, equitable distribution of scarce resources (such as access to life support equipment, staff time, and termination or withdrawal of care). [. . .] In case of limited supply of life saving interventions like mechanical ventilators, the decision of healthcare provider should be guided by the principle of first come first served and chances of survival based on the severity and reversibility of organ damage. [. . .] Any COVID-19 patient who requires emergency surgical or other interventions should not be denied these emergency services at any health facility, denying the service amounts to stigmatization.^19^

Our findings that chances of survival are likely included in prioritising COVID-19 patients align with FMOH’s guidelines. On the contrary, some of our findings seem to contradict it, including that at least some health professionals might de-prioritize older patients with COVID-19 when they have a better chance of survival than younger patients. Thus, as our results suggest, health professionals seem to use variable criteria when triaging patients, and some of these criteria might not always be consistent with mainstream ethical principles and guidelines. Further discussion of the knowledge and usage of ethical guidelines among health professionals is beyond our study. However, this contradiction implies that there could be gaps in the implementation of international as well as national ethical principles and guidelines among health professionals and that ethics training is critical to acquaint health professionals with existing treatment manuals and ethical guidelines.

### Limitations

Even though this study adds interesting insights into challenges to the healthcare system and ethical discussions concerning aged care in times of a global health crisis, it is not without limitations. One limitation is that due to the pandemic’s workload among health professionals and the risk of COVID-19 infection among older patients, we were not able to follow participants over time and do repeat interviews. Conducting serial interviews might have strengthened the rapport with participants and helped get more perspectives on the research question.^90^ However, the interviews were conducted by a trained and experienced researcher, and we believe that there was good rapport to get data rich enough to address the objective of the study. Moreover, as noted by Braun and Clarke,^53^our analysis and reporting in this study might have been influenced by our perspectives, especially given the exploratory qualitative approach employed, which lacks strict theoretical guidance. However, to reduce this risk, all authors reflected on the themes and critically reviewed the manuscript starting from the early draft. An additional limitation lies in the fact that we did not include informal or family care providers of older patients due to time and resource limitations mainly resulting from the pandemic. This might have affected the chances of getting more perspectives on the research objective. Lastly, none of the older adult participants reported having been tested COVID-positive. This might be due to different reasons such as low practice of covid testing in the country, fear of disclosing COVID infection history. Although the COVID status of participants was not part of the study’s objective, having participants with COVID infection would have added additional interesting perspectives.

## Conclusion

The unprecedented COVID-19 pandemic has brought health and wider socio-economic consequences in low-income countries like Ethiopia, and the most affected by these consequences are older adults. This study sheds more light on these challenges by demonstrating how the pandemic has affected the health and care of older adults in Ethiopia and illuminating ethical challenges this could give rise to, such as lack of healthcare access, health inequalities, unfair distribution of health resources, and ageism. Further research could include informal care providers and study ethical issues with making decisions that affect the health of older adults during a public health crisis. The findings of this study can inform preparedness and policy efforts tailored to efficiently address the health needs of older adults being affected by current and future global pandemics. More education and medical ethics training in the care of older patients can also assist health practitioners in Ethiopia to address ethical dilemmas in relation to priority setting that can affect the lives of older patients.

## Supplemental Material

sj-docx-1-smo-10.1177_20503121241263305 – Supplemental material for “I was afraid to go to the hospital”: A qualitative analysis and ethical implications of the impacts of COVID-19 on the health and medical care of older adults in Ethiopia Supplemental material, sj-docx-1-smo-10.1177_20503121241263305 for “I was afraid to go to the hospital”: A qualitative analysis and ethical implications of the impacts of COVID-19 on the health and medical care of older adults in Ethiopia by Kirubel Manyazewal Mussie, Jenny Setchell, Mirgissa Kaba and Bernice Simone Elger in SAGE Open Medicine

sj-docx-2-smo-10.1177_20503121241263305 – Supplemental material for “I was afraid to go to the hospital”: A qualitative analysis and ethical implications of the impacts of COVID-19 on the health and medical care of older adults in Ethiopia Supplemental material, sj-docx-2-smo-10.1177_20503121241263305 for “I was afraid to go to the hospital”: A qualitative analysis and ethical implications of the impacts of COVID-19 on the health and medical care of older adults in Ethiopia by Kirubel Manyazewal Mussie, Jenny Setchell, Mirgissa Kaba and Bernice Simone Elger in SAGE Open Medicine
