# Supplementary material for: “I was afraid to go to the hospital”: A qualitative analysis and ethical implications of the impacts of COVID-19 on the health and medical care of older adults in Ethiopia
Source: SAGE Open Med. 2024 Jul 30;12:20503121241263305. doi: 10.1177/20503121241263305 (PMC11292689; doi:10.1177/20503121241263305)
Supplement: sj-docx-2-smo-10.1177_20503121241263305 – Supplemental material for “I was afraid to go to the hospital”: A qualitative analysis and ethical implications of the impacts of COVID-19 on the health and medical care of older adults in Ethiopia [file sj-docx-2-smo-10.1177_20503121241263305.docx]

**Summary of the interview guides and probes**

**Interview guide for older adults**

1. Introduction and demographic information

- Please tell me about yourself, including your age, work or activities you do, family condition, etc?

1. Getting medical care in the time of COVID-19 pandemic

- How would you describe your health condition in general?
- Can you tell me what you know or hear about corona?
- How is your life in general after corona came and in what ways, if any, has it been affected by the pandemic?
- Can you tell me about your last visit, after the pandemic started, to a health care facility? How was it?
- How comfortable are you visiting health care facilities during the pandemic and what reasons could explain the level of your comfort?
- How do you describe the accessibility and quality of the health care services for you or other older patients getting medical care during the pandemic?
- Have you ever been diagnosed with and treated from corona? If so, could you explain the health consequences and your treatment experience?
- Can you comment on how the emergence of corona could relate to the ways health professionals treat older patients?

**Interview guide for health professionals**

1. Introduction and demographic information

- Please tell me about yourself, including your age, qualification, work experience, etc.

1. Experience of providing medical care for older patients during the pandemic?

- What are the differences in general, if any, between treating a younger patient and an older patient?
- What special resources or skills are needed to treat an older patient?
- How has the pandemic affected the availability and/or quality of health resources in health care facilities?
- How has the pandemic affected the ways you or other health professionals give health care services for older patients?
- How do you describe the older patient flow in your hospital after the pandemic has come, and especially if you compare it with the trend before?

1. The experience of treating older patients with COVID-19

- How do you describe the health conditions of older patients with COVID-19?
- How do you describe the treatment results or survival conditions of older patients with COVID-19, especially if you compare it with younger patients?
- Have you ever been in a situation where you had to prioritise among COVID-19 patients in general? If so, what were your reasons for doing that?
- How adequate are the resources to treat older patients with COVID-19?
- What do/would you do when you have limited health resources and you need to prioritise between an older and younger COVID-19 patients? What would be the reasons for your choice?
